# Hantavirus Pulmonary Syndrome, United States, 1993–2009

**DOI:** 10.3201/eid1707.101306

**Published:** 2011-07

**Authors:** Adam MacNeil, Thomas G. Ksiazek, Pierre E. Rollin

**Affiliations:** Author affiliations: Centers for Disease Control and Prevention, Atlanta, Georgia, USA (A. MacNeil, T.G. Ksiazek, P.E. Rollin);; University of Texas Medical Branch, Galveston, Texas, USA (T.G. Ksiazek)

## MEDSCAPE CME

Medscape, LLC is pleased to provide online continuing medical education (CME) for this journal article, allowing clinicians the opportunity to earn CME credit.

This activity has been planned and implemented in accordance with the Essential Areas and policies of the Accreditation Council for Continuing Medical Education through the joint sponsorship of Medscape, LLC and Emerging Infectious Diseases. Medscape, LLC is accredited by the ACCME to provide continuing medical education for physicians.

Medscape, LLC designates this Journal-based CME activity for a maximum of 1 *AMA PRA Category 1 Credit(s)*^TM^. Physicians should claim only the credit commensurate with the extent of their participation in the activity.

All other clinicians completing this activity will be issued a certificate of participation. To participate in this journal CME activity: (1) review the learning objectives and author disclosures; (2) study the education content; (3) take the post-test and/or complete the evaluation at www.medscape.org/journal/eid; (4) view/print certificate.

**Release date: June 24, 2011; Expiration date: June 24, 2012**

## Learning Objectives

Upon completion of this activity, participants will be able to:

- Evaluate the epidemiology of hantavirus infection
- Distinguish the region in the United States with the highest prevalence of hantavirus pulmonary syndrome (HPS)
- Analyze the prognosis of HPS
- Identify factors associated with a higher risk for mortality in cases of HPS.

## Editor

**Thomas Gryczan, **Technical Writer/Editor, *Emerging Infectious Diseases. Disclosure: Thomas Gryczan has disclosed no relevant financial relationships.*

## CME AUTHOR

**Charles P. Vega, MD**, Associate Professor; Residency Director, Department of Family Medicine, University of California, Irvine. *Disclosure: Charles P. Vega, MD, has disclosed no relevant financial relationships.*

## AUTHORS

*Disclosures:* ***Adam MacNeil, PhD, MPH; Thomas G. Ksiazek, DVM, PhD;*** *and* ***Pierre E. Rollin, MD,*** *have disclosed no relevant financial relationships.*
