# Supplementary material for: Hantavirus Pulmonary Syndrome, United States, 1993–2009
Source: Emerg Infect Dis. 2011 Jul;17(7):1195–201. doi: 10.3201/eid1707.101306 (PMC3321561; doi:10.3201/eid1707.101306)
Supplement: Related texts

# Research article: Hantavirus Pulmonary Syndrome, United States, 1993–2009

**DOI:** 10.3201/eid1707.101306

**Authors:** Adam MacNeil, Thomas G. Ksiazek, Pierre E. Rollin

**Affiliations:** Author affiliations: Centers for Disease Control and Prevention, Atlanta, Georgia, USA (A. MacNeil, T.G. Ksiazek, P.E. Rollin);; University of Texas Medical Branch, Galveston, Texas, USA (T.G. Ksiazek)

## Abstract

Hantavirus pulmonary syndrome (HPS) is a severe respiratory illness identified in 1993. Since its identification, the Centers for Disease Control and Prevention has obtained standardized information about and maintained a registry of all laboratory-confirmed HPS cases in the United States. During 1993–2009, a total of 510 HPS cases were identified. Case counts have varied from 11 to 48 per year (case-fatality rate 35%). However, there were no trends suggesting increasing or decreasing case counts or fatality rates. Although cases were reported in 30 states, most cases occurred in the western half of the country; annual case counts varied most in the southwestern United States. Increased hematocrits, leukocyte counts, and creatinine levels were more common in HPS case-patients who died. HPS is a severe disease with a high case-fatality rate, and cases continue to occur. The greatest potential for high annual HPS incidence exists in the southwestern United States.

In May 1993, a series of cases of an acute illness associated with rapid development of respiratory failure were noted in the Four Corners region of the United States. Surveillance initiated in the area identified 24 cases of compatible illness that had occurred in New Mexico, Arizona, Colorado, and Utah since December 1992; the case-fatality rate was 50%. Preliminary serologic data for case-patients suggested infection with an unknown virus in the family *Bunyaviridae* and genus *Hantavirus* (*1*). This observation was surprising, given that hantaviruses had not been associated with any human diseases in North or South America at that time, and the only known clinical syndrome associated with hantaviruses, hemorrhagic fever with renal syndrome, did not have a predominantly respiratory involvement. However, nucleic acid sequence from a novel hantavirus was rapidly identified in tissue samples of multiple patients, and similarly from deer mice (*Peromyscus maniculatus*) trapped near the residence of cases, implicating a novel hantavirus as the cause of the disease (*2*). Additional serologic and molecular data from case-patients and results of trapping studies in the Four Corners region supported these conclusions (*3**,**4*).

Since its identification in 1993, hantavirus cardiopulmonary syndrome (HPS) and numerous New World hantavirus species have been described across a wide geographic range of North, Central, and South America (*5*). In the United States, most HPS cases are likely caused by Sin Nombre virus (*6*), the virus responsible for the initially identified HPS cases. Other HPS-associated viruses include New York and Monongahela viruses (mice of the genus *Peromyscus* are reservoirs), associated with HPS in the eastern United States (*7**–**9*), Bayou virus, found in the southeastern United States (*Oligoryzomys palustris* rice rats are reservoirs) (*10**–**12*), and Black Creek Canal virus (*Sigmodon hispidus* cotton rats are reservoirs), which was associated with 1 case of HPS in Florida (*13**,**14*).

Hantaviruses are believed to be transmitted by inhalation of rodent secretions and excreta, or possibly through direct contact with an infected rodent. Although clusters of human cases have been identified in the United States, no evidence exists of human-to-human or nosocomial transmission of hantaviruses in North America (*15**,**16*) Infrequent but clear instances of human-to-human transmission of Andes virus in Argentina and Chile have been documented (*17**–**19*).

The incubation period of HPS is believed to range from 1 to 5 weeks (*20*). HPS typically begins with a prodromal syndrome, and common symptoms include fever, myalgias, headache, and nausea/vomiting (*21**,**22*). After the prodrome, the hallmark of HPS is rapid onset of a severe pulmonary illness, often involving hypoxia, pulmonary edema, and myocardial depression (*22**–**25*). Death typically occurs rapidly after hospitalization (*21*) and often as the result of cardiogenic shock (*25*). In this report, we evaluate the epidemiologic and clinical characteristics of all known laboratory-confirmed cases of HPS in the United States during 1993–2009.

## Materials and Methods

After identification of HPS in 1993, the Viral Special Pathogens Branch at the Centers for Disease Control and Prevention (Atlanta, GA, USA) developed and maintained a registry of confirmed HPS cases in the United States. A clinically confirmed case of HPS is defined as 1) a febrile illness characterized by bilateral diffuse interstitial edema that may radiographically resemble acute respiratory distress syndrome (ARDS), with respiratory compromise requiring supplemental oxygen developing <72 hours after hospitalization, and occurring in a previously healthy person, or an unexplained respiratory illness resulting in death, with an autopsy examination demonstrating pulmonary noncardiogenic edema without an identifiable cause; and 2) laboratory evidence of hantavirus infection by detection of hantavirus-specific immunoglobulin M or increasing titers of hantavirus-specific immunoglobulin G, detection of hantavirus-specific RNA sequence by PCR in clinical specimens, or detection of hantavirus antigen by immunohistochemical analysis (*26*).

Information for the registry, including demographic, geographic, outcome, and (if possible) basic clinical data, is obtained by using a case-report form. Although many laboratory diagnoses are not made at the Centers for Disease Control and Prevention, case-report forms were reviewed to verify hantavirus laboratory diagnostics. Additionally, since 1995, HPS has been a nationally reportable disease in the United States; thus, parallel surveillance for HPS is conducted through the National Notifiable Diseases Surveillance System (*26*). To ensure completeness of the registry, we attempted to acquire case-report forms for all HPS cases reported to the National Notifiable Diseases Surveillance System. For all cases, we attempted to acquire qualitative data (yes or no) regarding certain clinical signs and symptoms, and laboratory values (fever, thrombocytopenia, increased hematocrit, increased creatinine levels, leukocyte counts, chest radiograph showing unexplained bilateral infiltrates or suggestive of ARDS, requirement of supplemental oxygen, and whether the patient was intubated). For a small number of patients for whom incomplete qualitative data were available, but for whom laboratory values were available, we assigned qualitative values on the basis of described clinical cutoff values (*24*).

## Results

### Number of HPS Cases and Demographics

During 1993–2009, we identified 510 laboratory-confirmed cases of HPS in the United States; 30 cases that occurred before 1993 had been retrospectively identified (*27**–**29*). HPS is primarily a disease of adults; most cases were in persons 20–50 years of age (mean age 38 years) (Table 1); 7% of cases occurred in children <16 years of age. Among HPS cases, 64% occurred in male patients. Most cases occurred in white (78%) or American Indian/Native American (20%) persons, and 21% of case-patients reported their ethnicity as Hispanic.

**Table 1:** Demographic characteristics of HPS case-patients, United States, 1993–2009*

Characteristic	No. (%) cases	Outcome	
		CFR, %	p value†
Age, y, n = 508			0.992
<10	5 (1)	20	
10–19	56 (11)	36	
20–29	116 (23)	36	
30–39	105 (21)	37	
40–49	101 (20)	33	
50–59	69 (14)	35	
60–69	48 (9)	33	
>70	8 (2)	38	
Sex, n = 510			0.180
M	324 (64)	33	
F	186 (36)	39	
Race, n = 492			0.284
White	383 (78)	33	
American Indian or Native American	96 (20)	43	
Black	8 (2)	38	
Asian or Pacific Islander	5 (1)	60	
Ethnicity, n = 393			0.637
Non-Hispanic	312 (79)	37	
Hispanic	81 (21)	40	

*HPS, hantavirus pulmonary syndrome; CFR, case-fatality rate. †χ^2^ p value for testing the difference in proportion of HPS case-patients who died between categories for each demographic characteristic.

### Temporal and Geographic Characteristics of Cases

The largest annual number of HPS cases registered (n = 48) occurred in 1993 during the initial investigation. Since that time, annual case counts have varied considerably from year to year (Figure 1); counts have ranged from 11 to 45 cases/year (mean 30 cases/year). No significant trend was observed in increasing or decreasing case counts from after 1993 (p = 0.400, by general linear model). From a population perspective, HPS is rare in the United States; the annual incidence has ranged from 0.04 to 0.19 cases/million persons on the basis of a US Census Bureau estimate of the US population on July 1 of each respective year (www.census.gov). HPS displayed a strong seasonal distribution; the maximum number of cases occurred in May, June, and July, and the minimum occurred in December, January, and February (Figure 2).

**Figure 1:** Annual number of cases (bars) of and case-fatality rate (line) for hantavirus pulmonary syndrome, United States, 1993–2009.

**Figure 2:** Cumulative number of hantavirus pulmonary syndrome cases by month of onset, United States, 1993–2009.

We identified the probable geographic location of rodent exposure (at least to the state level) for 471 cases. Probable exposures occurred in 30 US states; most cases occurred in the western United States To examine trends in HPS occurrence, we grouped states into 4 regions on the basis of geography and hantavirus species present: Southwest (Arizona, California, Colorado, New Mexico, Nevada, Utah); Northwest (Idaho, Montana, Oregon, Washington, and Wyoming); Midwest (Illinois, Indiana, Iowa, Kansas, Louisiana, Minnesota, North Dakota, Nebraska, Oklahoma, South Dakota, Texas, and Wisconsin); and East (Florida, Maryland, North Carolina, New York, Pennsylvania, Virginia, and West Virginia) (Table 2).

**Table 2:** HPS case-patients by geographic region, United States, 1993–2009*

Region	States†	Hantavirus species present in region	No. case-patients who died/ total no. case-patients (CFR, %)‡
Southwest	Arizona, California, Colorado, New Mexico, Nevada, Utah	Sin Nombre virus	92/273 (34)
Northwest	Idaho, Montana, Oregon, Washington, Wyoming	Sin Nombre virus	35/101 (35)
Midwest	Illinois, Indiana, Iowa, Kansas, Louisiana, Minnesota, North Dakota, Nebraska, Oklahoma, South Dakota, Texas, Wisconsin	Sin Nombre virus, Bayou virus	33/84 (39)
East	Florida, Maryland, North Carolina, New York, Pennsylvania, Virginia, West Virginia	New York virus, Monongahela virus, Black Creek Canal virus	5/12 (43)

*HPS, hantavirus pulmonary syndrome; CFR, case-fatality rate. †Only states with >1 probable rodent exposure–related HPS case are shown. ‡There were no significant differences in case-fatality rates between geographic regions (p = 0.773 by χ^2^ test).

We assessed temporal trends in HPS for each of these regions (with the exception of the East because there were only 12 cases) (Figure 3, panel A). Overall case counts were relatively stable across the 17-year period in the Northwest (mean ± SD 5.9 ± 2.1 cases/year) and Midwest (mean ± SD 4.9 ± 2.8 cases/year). In contrast, case counts in the Southwest tended to fluctuate to a higher degree (mean ± SD 16.1 ± 9.8 cases/year). Overall variance in annual case counts was significantly higher in the Southwest than in the Northwest (F-statistic p<0.001) or the Midwest (F-statistic p<0.001). When combined with annual HPS case counts for the entire United States, peaks in HPS case counts in the Southwest corresponded directly with peaks for the entire country. This finding, in conjunction with stable case counts in the Northwest and Midwest, demonstrates that annual variability in HPS in the United States is primarily driven by fluctuations in number of HPS cases in the southwestern United States.

**Figure 3:** Annual number of cases of hantavirus pulmonary syndrome (HPS) (A) and percentage of cases by month of onset (B) by geographic region of probable HPS exposure, United States, 1993–2009.

We assessed seasonality of HPS by geographic region (Figure 3, panel B). Similar to aggregate trends for the entire United States, HPS displayed a clear seasonal trend in the Midwest, Northwest, and Southwest. In contrast to the Midwest and Northwest, in which the highest proportion of cases occurred in May and decreased in the summer months, HPS cases peaked 2 months later (in July) in the Southwest.

### Clinical Characteristics and Case-Fatality Rates

The overall case-fatality rate was 35%. Deaths varied noticeably from year to year (Figure 1); however, no temporal trend in deaths was observed (p = 0.307, by Cochran-Armitage trend test). Additionally, case-fatality rates were similar across demographic characteristics; no differences in case-fatality rates were noted for age groups, or by sex, race, or ethnicity (Table 1). Similarly, from a geographic standpoint, case-fatality rates did not differ between geographic regions (p = 0.773, by χ^2^ test) (Table 2). The mean time from onset of symptoms to death was 6.4 days (median 5 days).

As described (*21**,**22*), HPS case-patients had a severe respiratory illness (most persons had chest radiographs showing unexplained bilateral infiltrates or suggestive of ARDS, and required supplemental oxygen) and thrombocytopenia (Table 3). Other common findings included fever >101°F and increased hematocrits, creatinine levels, and leukocyte counts. Increased hematocrits, creatinine levels, and leukocyte counts; requirement for supplemental oxygen; and necessity for intubation were all associated with death of a patient. Additionally, although nearly all HPS case-patients had thrombocytopenia, platelet counts (lowest measured value during illness) were significantly lower among patients who died than among patients who survived (median platelet count in persons who died 33,500 cells/mL vs. median in persons who survived 51,500 cells/mL; p<0.001 by Wilcoxon rank-sum test), for 278 persons for whom data were available.

**Table 3:** Clinical characteristics of HPS case-patients, United States, 1993–2009*

Characteristic	No. patients reporting characteristic/no. patients for whom data were available for that characteristic (%)			p value†
	All	Died	Survived	
Fever	356/397 (90)	116/132 (88)	239/264 (91)	0.414
Thrombocytopenia‡	405/418 (97)	131/134 (98)	273/283 (96)	0.477
Increased hematocrit‡	261/389 (67)	103/130 (79)	157/258 (61)	<0.001
Increased creatinine level‡	120/286 (42)	54/93 (58)	66/193 (34)	<0.001
Increased leukocyte count‡	169/234 (72)	72/83 (87)	96/150 (64)	<0.001
Chest radiograph showing unexplained bilateral infiltrates or suggestive of ARDS	390/406 (96)	135/141 (96)	255/265 (96)	0.812
Need for supplemental oxygen	390/405 (96)	134/135 (99)	255/269 (95)	0.025
Intubation of patient	265/430 (62)	129/139 (93)	135/290 (47)	<0.001

*HPS, hantavirus pulmonary syndrome; ARDS, acute respiratory distress syndrome. †By χ^2^ test for difference in the proportion of HPS case-patients with a clinical characteristic who died and those who survived. ‡Based on qualitative data (yes or no) regarding clinical characteristic from case investigation form. For a small number of case-patients for whom qualitative data were incomplete, but for whom laboratory values were available, qualitative values were assigned on the basis of described clinical cutoff values (*24*).

## Discussion

In 1996, Khan et al. published a description of the first 100 cases of HPS identified in the United States (*21*). Some aspects of the epidemiology of HPS in the United States have since been discussed in the peer-reviewed literature. However, no studies have provided a comprehensive evaluation of the epidemiology of HPS in the United States. By maintaining a registry and obtaining information in standardized manner, we were able to evaluate the epidemiologic characteristics of HPS in >500 cases over 17 years of data collection. Although HPS is a nationally reportable disease in the United States, maintenance of our registry has enabled us to obtain more detailed and standardized information on HPS than otherwise possible through other national surveillance mechanisms.

HPS is often characterized as an emerging infectious disease. The discovery of a novel clinical syndrome and associated virus might constitute emergence from a public health perspective. However, several lines of evidence indicate the epidemiology of HPS constitutes that of an endemic or sporadic disease. First, although overall numbers clearly vary from year to year, our data demonstrate continual occurrence of HPS since 1993 without a trend toward an increasing or decreasing number of cases. Additionally, HPS cases before 1993 in the United States (*27**–**29*), possibly as far back as 1959 (*30*), and rodents infected with hantaviruses (*6**,**31*) have been identified retrospectively. Similarly, the evolutionary history of hantavirus species in the United States appears to have been closely linked with that of their primary rodent host species and to persist over time among these host species (*32*), consistent with the notion that hantaviruses are not newly emergent in the United States. Finally, HPS and wide distribution of associated hantavirus species across most of the New World do not support recent emergence of a novel pathogenic virus.

The distribution of rodent reservoirs of pathogenic hantaviruses covers the entire mainland United States (*33*). During 1993–2009, HPS cases were associated with probable rodent exposures in 31 US states. However, in contrast to the wide distribution of rodent reservoirs, HPS is clearly more common in the western United States; only a small proportion (<3%) of cases are associated with exposures in the eastern United States. Although the virus species responsible for HPS is not typically assessed in diagnostic testing, it is likely that most cases of HPS in the United States are caused by Sin Nombre virus because of the western distribution of the reservoir host of this virus, the deer mouse, in comparison with reservoir hosts of other pathogenic hantavirus species, which are found primarily in the central and eastern United States.

Our examination of the epidemiology of HPS on the basis of geographic region has obvious limitations. For instance, state boundaries do not necessarily represent boundaries of ecosystems or distribution of reservoirs of different pathogenic hantavirus species. We noted major conclusions from this approach. First, we examined the hypothesis that hantavirus species may differ in their pathogenic potential. Although the overall number of HPS cases in the eastern United States was small (n = 12) and infections were potentially caused by multiple hantavirus species in the East and Midwest regions, our data do not suggest a difference in pathogenicity between hantavirus species endemic to the United States. Furthermore, systematic viral genotyping is needed to conclusively address the hypothesis that hantaviruses in the United States may differ in their pathogenic potential in humans. Second, annual numbers of HPS cases in the Northwest and Midwest were relatively consistent, whereas annual HPS case counts in the Southwest were significantly more variable, and peak years of HPS in the Southwest corresponded with high overall case-count years in the United States. These findings suggest greater potential for increases in HPS in the southwestern United States than in other regions of the country.

The HPS case-fatality rate in the United States was 35% during 1993–2009. No antiviral treatment is available for HPS, and we did not observe a trend in the case-fatality rate for HPS over time. No demographic factors were associated with deaths caused by HPS outcomes. The apparent rarity of HPS in younger persons is notable. However, similar case-fatality rates for HPS across age groups and results of case studies of HPS in children (*34**,**35*) indicate that severity of HPS is likely similar in adults and younger persons. We have limited data about the relationship between concurrent conditions and HPS outcome. However, similar case-fatality rates for HPS across age groups (particularly that the case-fatality rate remains similar in older persons) does not support the notion that underlying health conditions are the primary determinant of disease outcome. The actual level of virus exposure at the time of infection may also be a major determinant of disease severity. A recent study reported smoking as a significant risk factor for Puumala virus (genera *Hantavirus*) infection in Finland (*36*). We believe this finding warrants a study in the United States to determine whether smoking might increase the likelihood of development or the overall severity of HPS.

HPS is characterized by the rapid onset of a severe respiratory disease. Virtually all patients with laboratory-confirmed HPS in our registry for whom clinical data were available required supplemental oxygen (96%) and had a chest radiograph showing unexplained bilateral infiltrates or suggestive of ARDS (96%). Although thrombocytopenia is a common symptom of HPS, lowest platelet counts were lower in fatal HPS cases. Similarly, increased hematocrits, creatinine levels, and leukocyte counts occurred in a higher proportion in HPS case-patients who died. Although similar clinical findings have been reported for smaller case studies (*21**–**24**,**37*), our data demonstrate the role of these factors in predicting the outcome of HPS. We also noted similar associations between outcomes in patients who died and the requirement for supplementary oxygen and intubation. These 2 variables represent clinical procedures and thus would be expected to be more common in severe HPS cases. Although we do not have any data about the proportion of case-patients who received extracorpeal membrane oxygenation, some studies suggest that this procedure might improve the prognosis for severe HPS (*38**,**39*).

Because of the centralized and passive nature of data collection, our methods have some limitations. Data collection was limited to a short, standardized case investigation form; thus, we were unable to collect detailed clinical information and verify clinical information (such as radiographic findings). In addition, clinical aspects of our surveillance data were limited to a small number of specific criteria. Other investigators have reported signs, symptoms, radiographic characteristics, and pathologic features of HPS in greater detail (*21**–**24**,**40*). Studies have also demonstrated cardiopulmonary depression, and resulting cardiogenic shock, as a major pathologic aspect of HPS, particularly in patients who died (*25*). In addition, because of the passive nature of HPS surveillance, we may have missed some HPS cases in the United States. However, through continued outreach with state health departments and the ability to cross-check HPS cases with those reported through the National Notifiable Diseases Surveillance System, we have attempted to minimize the number of HPS cases that might go unregistered. However, frequently updated HPS case counts and geographic data are available (www.cdc.gov/hantavirus).

Despite its rarity, HPS continues to occur in the United States. With a case-fatality rate of 35%, HPS remains 1 of the most severe infectious diseases endemic to the United States.

## References

[1] Centers for Disease Control and Prevention. Outbeak of acute illness—southwestern United States, 1993. MMWR Morb Mortal Wkly Rep. 1993;42:421–4.8502213

[2] Nichol ST, Spiropoulou CF, Morzunov S, Rollin PE, Ksiazek TG, Feldmann H, Genetic identification of a hantavirus associated with an outbreak of acute respiratory illness. Science. 1993;262:914–7. 10.1126/science.82356158235615

[3] Ksiazek TG, Peters CJ, Rollin PE, Zaki S, Nichol S, Spiropoulou C, Identification of a new North American hantavirus that causes acute pulmonary insufficiency. Am J Trop Med Hyg. 1995;52:117–23.787243710.4269/ajtmh.1995.52.117

[4] Childs JE, Ksiazek TG, Spiropoulou CF, Krebs JW, Morzunov S, Maupin GO, Serologic and genetic identification of *Peromyscus maniculatus* as the primary rodent reservoir for a new hantavirus in the southwestern United States. J Infect Dis. 1994;169:1271–80. 10.1093/infdis/169.6.12718195603

[5] Peters CJ, Khan AS. Hantavirus pulmonary syndrome: the new American hemorrhagic fever. Clin Infect Dis. 2002;34:1224–31. 10.1086/33986411941549

[6] Monroe MC, Morzunov SP, Johnson AM, Bowen MD, Artsob H, Yates T, Genetic diversity and distribution of *Peromyscus*-borne hantaviruses in North America. Emerg Infect Dis. 1999;5:75–86. 10.3201/eid0501.99010910081674PMC2627704

[7] Song JW, Baek LJ, Gajdusek DC, Yanagihara R, Gavrilovskaya I, Luft BJ, Isolation of pathogenic hantavirus from white-footed mouse (*Peromyscus leucopus*). Lancet. 1994;344:1637. 10.1016/S0140-6736(94)90430-87984010

[8] Hjelle B, Lee SW, Song W, Torrez-Martinez N, Song JW, Yanagihara R, Molecular linkage of hantavirus pulmonary syndrome to the white-footed mouse, *Peromyscus leucopus*: genetic characterization of the M genome of New York virus. J Virol. 1995;69:8137–41.749433710.1128/jvi.69.12.8137-8141.1995PMC189769

[9] Rhodes LV III, Huang C, Sanchez AJ, Nichol ST, Zaki SR, Ksiazek TG, Hantavirus pulmonary syndrome associated with Monongahela virus, Pennsylvania. Emerg Infect Dis. 2000;6:616–21. 10.3201/eid0606.00061011076720PMC2640920

[10] Morzunov SP, Feldmann H, Spiropoulou CF, Semenova VA, Rollin PE, Ksiazek TG, A newly recognized virus associated with a fatal case of hantavirus pulmonary syndrome in Louisiana. J Virol. 1995;69:1980–3.785354510.1128/jvi.69.3.1980-1983.1995PMC188821

[11] Ksiazek TG, Nichol ST, Mills JN, Groves MG, Wozniak A, McAdams S, Isolation, genetic diversity, and geographic distribution of Bayou virus (Bunyaviridae: hantavirus). Am J Trop Med Hyg. 1997;57:445–8.934796110.4269/ajtmh.1997.57.445

[12] Torrez-Martinez N, Bharadwaj M, Goade D, Delury J, Moran P, Hicks B, Bayou virus-associated hantavirus pulmonary syndrome in eastern Texas: identification of the rice rat, *Oryzomys palustris*, as reservoir host. Emerg Infect Dis. 1998;4:105–11. 10.3201/eid0401.9801159452404PMC2627679

[13] Rollin PE, Ksiazek TG, Elliott LH, Ravkov EV, Martin ML, Morzunov S, Isolation of Black Creek Canal virus, a new hantavirus from *Sigmodon hispidus* in Florida. J Med Virol. 1995;46:35–9. 10.1002/jmv.18904601087623004

[14] Khan AS, Gaviria M, Rollin PE, Hlady WG, Ksiazek TG, Armstrong LR, Hantavirus pulmonary syndrome in Florida: association with the newly identified Black Creek Canal virus. Am J Med. 1996;100:46–8. 10.1016/S0002-9343(96)90010-88579086

[15] Vitek CR, Breiman RF, Ksiazek TG, Rollin PE, McLaughlin JC, Umland ET, Evidence against person-to-person transmission of hantavirus to health care workers. Clin Infect Dis. 1996;22:824–6. 10.1093/clinids/22.5.8248722939

[16] Wells RM, Young J, Williams RJ, Armstrong LR, Busico K, Khan AS, Hantavirus transmission in the United States. Emerg Infect Dis. 1997;3:361–5. 10.3201/eid0303.9703149284382PMC2627629

[17] Enría D, Padula P, Segura EL, Pini N, Edelstein A, Posse CR, Hantavirus pulmonary syndrome in Argentina. Possibility of person to person transmission. Medicina (B Aires). 1996;56:709–11.9284576

[18] Chaparro J, Vega J, Terry W, Vera JL, Barra B, Meyer R, Assessment of person-to-person transmission of hantavirus pulmonary syndrome in a Chilean hospital setting. J Hosp Infect. 1998;40:281–5. 10.1016/S0195-6701(98)90304-89868619

[19] Martinez VP, Bellomo C, San Juan J, Pinna D, Forlenza R, Elder M, Person-to-person transmission of Andes virus. Emerg Infect Dis. 2005;11:1848–53.1648546910.3201/eid1112.050501PMC3367635

[20] Young JC, Hansen GR, Graves TK, Deasy MP, Humphreys JG, Fritz CL, The incubation period of hantavirus pulmonary syndrome. Am J Trop Med Hyg. 2000;62:714–7.1130406110.4269/ajtmh.2000.62.714

[21] Khan AS, Khabbaz RF, Armstrong LR, Holman RC, Bauer SP, Graber J, Hantavirus pulmonary syndrome: the first 100 US cases. J Infect Dis. 1996;173:1297–303. 10.1093/infdis/173.6.12978648200

[22] Duchin JS, Koster FT, Peters CJ, Simpson GL, Tempest B, Zaki SR, Hantavirus pulmonary syndrome: a clinical description of 17 patients with a newly recognized disease. The Hantavirus Study Group. N Engl J Med. 1994;330:949–55. 10.1056/NEJM1994040733014018121458

[23] Nolte KB, Feddersen RM, Foucar K, Zaki SR, Koster FT, Madar D, Hantavirus pulmonary syndrome in the United States: a pathological description of a disease caused by a new agent. Hum Pathol. 1995;26:110–20. 10.1016/0046-8177(95)90123-X7821907

[24] Zaki SR, Greer PW, Coffield LM, Goldsmith CS, Nolte KB, Foucar K, Hantavirus pulmonary syndrome. Pathogenesis of an emerging infectious disease. Am J Pathol. 1995;146:552–79.7887439PMC1869168

[25] Hallin GW, Simpson SQ, Crowell RE, James DS, Koster FT, Mertz GJ, Cardiopulmonary manifestations of hantavirus pulmonary syndrome. Crit Care Med. 1996;24:252–8. 10.1097/00003246-199602000-000128605797

[26] Centers for Disease Control and Prevention. Case definitions for infectious conditions under public health surveillance. MMWR Recomm Rep. 1997;46:1–55.9148133

[27] Zaki SR, Albers RC, Greer PW, Coffield LM, Armstrong LR, Khan AS, Retrospective diagnosis of a 1983 case of fatal hantavirus pulmonary syndrome. Lancet. 1994;343:1037–8. 10.1016/S0140-6736(94)90156-27909065

[28] Zaki SR, Khan AS, Goodman RA, Armstrong LR, Greer PW, Coffield LM, Retrospective diagnosis of hantavirus pulmonary syndrome, 1978–1993: implications for emerging infectious diseases. Arch Pathol Lab Med. 1996;120:134–9.8712893

[29] Schwarcz SK, Shefer AM, Zaki SR. Retrospective diagnosis of a fatal case of the hantavirus pulmonary syndrome, 1980. West J Med. 1996;164:348–50.8732742PMC1303518

[30] Frampton JW, Lanser S, Nichols CR. Sin Nombre virus infection in 1959. Lancet. 1995;346:781–2. 10.1016/S0140-6736(95)91542-77658902

[31] Nerurkar VR, Song JW, Song KJ, Nagle JW, Hjelle B, Jenison S, Genetic evidence for a hantavirus enzootic in deer mice (*Peromyscus maniculatus*) captured a decade before the recognition of hantavirus pulmonary syndrome. Virology. 1994;204:563–8. 10.1006/viro.1994.15707941323

[32] Nichol S. Genetic analysis of hantaviruses and their host relationships. In: Saluzzo J, Dodet B, editors. Factors in the emergence and control of rodent-borne viral diseases: San Francisco: Elsevier; 1999. p. 99–109.

[33] Mills JN, Amman BR, Glass GE. Ecology of hantaviruses and their hosts in North America. Vector Borne Zoonotic Dis. 2010;10:563–74. 10.1089/vbz.2009.001819874190

[34] Ramos MM, Overturf GD, Crowley MR, Rosenberg RB, Hjelle B. Infection with Sin Nombre hantavirus: clinical presentation and outcome in children and adolescents. Pediatrics. 2001;108:E27. 10.1542/peds.108.2.e2711483837

[35] Centers for Disease Control and Prevention. Hantavirus pulmonary syndrome in five pediatric patients—four states, 2009. MMWR Morb Mortal Wkly Rep. 2009;58:1409–12.20032925

[36] Vapalahti K, Virtala AM, Vaheri A, Vapalahti O. Case–control study on Puumala virus infection: smoking is a risk factor. Epidemiol Infect. 2010;138:576–84. 10.1017/S095026880999077X19781114

[37] Koster F, Foucar K, Hjelle B, Scott A, Chong YY, Larson R, Rapid presumptive diagnosis of hantavirus cardiopulmonary syndrome by peripheral blood smear review. Am J Clin Pathol. 2001;116:665–72. 10.1309/CNWF-DC72-QYMR-M8DA11710682

[38] Crowley MR, Katz RW, Kessler R, Simpson SQ, Levy H, Hallin GW, Successful treatment of adults with severe hantavirus pulmonary syndrome with extracorporeal membrane oxygenation. Crit Care Med. 1998;26:409–14. 10.1097/00003246-199802000-000479468181

[39] Dietl CA, Wernly JA, Pett SB, Yassin SF, Sterling JP, Dragan R, Extracorporeal membrane oxygenation support improves survival of patients with severe hantavirus cardiopulmonary syndrome. J Thorac Cardiovasc Surg. 2008;135:579–84. 10.1016/j.jtcvs.2007.11.02018329474

[40] Ketai LH, Williamson MR, Telepak RJ, Levy H, Koster FT, Nolte KB, Hantavirus pulmonary syndrome: radiographic findings in 16 patients. Radiology. 1994;191:665–8.818404310.1148/radiology.191.3.8184043

# Research article: Hantavirus Pulmonary Syndrome, United States, 1993–2009

**DOI:** 10.3201/eid1707.101306

**Authors:** Adam MacNeil, Thomas G. Ksiazek, Pierre E. Rollin

**Affiliations:** Author affiliations: Centers for Disease Control and Prevention, Atlanta, Georgia, USA (A. MacNeil, T.G. Ksiazek, P.E. Rollin);; University of Texas Medical Branch, Galveston, Texas, USA (T.G. Ksiazek)

## Earning CME Credit

To obtain credit, you should first read the journal article. After reading the article, you should be able to answer the following, related, multiple-choice questions. To complete the questions and earn continuing medical education (CME) credit, please go to **www.medscape.org/journal/eid**. Credit cannot be obtained for tests completed on paper, although you may use the worksheet below to keep a record of your answers. You must be a registered user on Medscape.org. If you are not registered on Medscape.org, please click on the New Users: Free Registration link on the left hand side of the website to register. Only one answer is correct for each question. Once you successfully answer all post-test questions you will be able to view and/or print your certificate. For questions regarding the content of this activity, contact the accredited provider, CME@medscape.net. For technical assistance, contact CME@webmd.net. American Medical Association’s Physician’s Recognition Award (AMA PRA) credits are accepted in the US as evidence of participation in CME activities. For further information on this award, please refer to http://www.ama-assn.org/ama/pub/category/2922.html. The AMA has determined that physicians not licensed in the US who participate in this CME activity are eligible for *AMA PRA Category 1 Credits*™. Through agreements that the AMA has made with agencies in some countries, AMA PRA credit is acceptable as evidence of participation in CME activities. If you are not licensed in the US and want to obtain an AMA PRA CME credit, please complete the questions online, print the certificate and present it to your national medical association.

## Article Title: Hantavirus Pulmonary Syndrome, United States, 1993–2009

## CME Questions

**1. You are seeing a 50-year-old woman with a 2-day history of headache, myalgia, and fever to 39.5°C. While your differential diagnosis remains broad for this patient, what should you consider regarding the epidemiology of infection with hantavirus?**A. The majority of hantavirus pulmonary syndrome (HPS) cases in the United States are caused by the Sin Nombre virusB. Hantavirus is primarily transmitted via droplet secretions from human to humanC. The incubation period of HPS is usually less than 48 hoursD. Human fecal-oral transmission is the principal source of hantavirus**2. The prevalence of HPS is highest and most variable over time in which region of the United States?**A. Eastern regionB. Southern regionC. Midwest regionD. Southwest region**3. The patient from Question #1 develops HPS. What does the current study suggest regarding her prognosis?**A. The case-fatality rate was 35%B. Older age predicted a higher fatality rateC. Patients in the Southwest were most likely to die from HPSD. The mean time from symptom onset to death was 3.5 weeks**4. Based on the results of the current study, which of the following laboratory findings is associated with the most significant risk for mortality due to HPS?**A. Any thrombocytopeniaB. Reduced hematocritC. Elevated serum creatinineD. Reduced serum albumin

## Activity Evaluation

**Table:** 

1. The activity supported the learning objectives.				
Strongly Disagree				Strongly Agree
1	2	3	4	5
2. The material was organized clearly for learning to occur.				
Strongly Disagree				Strongly Agree
1	2	3	4	5
3. The content learned from this activity will impact my practice.				
Strongly Disagree				Strongly Agree
1	2	3	4	5
4. The activity was presented objectively and free of commercial bias.				
Strongly Disagree				Strongly Agree
1	2	3	4	5
